# Vitamin A metabolism is changed in donors after living-kidney transplantation: an observational study

**DOI:** 10.1186/1476-511X-10-231

**Published:** 2011-12-07

**Authors:** Andrea Henze, Jens Raila, Caroline Kempf, Petra Reinke, Anett Sefrin, Uwe Querfeld, Florian J Schweigert

**Affiliations:** 1 Institute of Nutritional Science, Department of Physiology and Pathophysiology, University of Potsdam, Nuthetal, Germany; 2 Department of Pediatric Nephrology, Charité Universitätsmedizin Berlin, Berlin, Germany; 3 Medical Department, Division of Nephrology and Intensive Care Medicine, Charité Universitätsmedizin Berlin, Berlin, Germany

**Keywords:** Donors, glomerular filtration rate, kidney transplantation, retinol, retinol-binding protein 4, transthyretin

## Abstract

**Background:**

The kidneys are essential for the metabolism of vitamin A (retinol) and its transport proteins retinol-binding protein 4 (RBP4) and transthyretin. Little is known about changes in serum concentration after living donor kidney transplantation (LDKT) as a consequence of unilateral nephrectomy; although an association of these parameters with the risk of cardiovascular diseases and insulin resistance has been suggested. Therefore we analyzed the concentration of retinol, RBP4, apoRBP4 and transthyretin in serum of 20 living-kidney donors and respective recipients at baseline as well as 6 weeks and 6 months after LDKT.

**Results:**

As a consequence of LDKT, the kidney function of recipients was improved while the kidney function of donors was moderately reduced within 6 weeks after LDKT. With regard to vitamin A metabolism, the recipients revealed higher levels of retinol, RBP4, transthyretin and apoRBP4 before LDKT in comparison to donors. After LDKT, the levels of all four parameters decreased in serum of the recipients, while retinol, RBP4 as well as apoRBP4 serum levels of donors increased and remained increased during the follow-up period of 6 months.

**Conclusion:**

LDKT is generally regarded as beneficial for allograft recipients and not particularly detrimental for the donors. However, it could be demonstrated in this study that a moderate reduction of kidney function by unilateral nephrectomy, resulted in an imbalance of components of vitamin A metabolism with a significant increase of retinol and RBP4 and apoRBP4 concentration in serum of donors.

## Background

Kidney transplantation is increasingly performed by living donation. Living donor kidney transplantation (LDKT) provides superior allograft function for the recipients [1] with a low risk of complications such as hypertension and chronic kidney disease (CKD) for donors. However, unilateral nephrectomy leads to a reduction of the donor's glomerular filtration rate (GFR) [2,3], which could result in altered renal clearance and catabolism of many substances, thereby potentially increasing the risk for metabolic disturbances after LDKT. Such metabolic disturbances could mediate an increased risk for cardiovascular disease (CVD), which is present even in early stages of CKD [4]. Among the substances with altered renal clearance are the compounds of the vitamin A transport complex, namely retinol (ROH), retinol-binding protein 4 (RBP4) and transthyretin (TTR). ROH represents the transport form of vitamin A and is specifically transported by RBP4 in the circulation. Furthermore, the ROH-RBP4 complex is non-covalently linked to the visceral protein TTR [5]. The kidneys are important for metabolism and catabolism of ROH, RBP4 and TTR [5-7] and serum concentrations of all three metabolites are increased in patients with reduced kidney function [8,9]. Especially RBP4 serum concentration has been shown to be closely associated with GFR in a reverse fashion [10,11]. Vitamin A is essential for numerous biological functions such as vision, cellular differentiation and immune defense [7]. However, elevated ROH as well as RBP4 serum concentrations have been associated with an increased risk of CVD [12-15]. Additionally, high levels of RBP4 have been linked to the development of insulin resistance [16]. Therefore, increased ROH and RBP4 serum concentrations in donors may increase their risk of CVD and insulin resistance post-nephrectomy.

Since very little is known so far about changes of ROH, RBP4 and TTR serum concentrations in kidney donors, the aim of the present study was to determine the changes of all three substances before and after transplantation in serum of donors and recipients as well as their association to estimated GFR (eGFR). Additionally, the relative amount of circulating apoRBP4 (RBP4 free from ROH) was determined, since apoRBP4 represents a physiologic signal for an increased hepatic holoRBP4 (RBP4 with ROH) secretion, which could theoretically lead to further increase of ROH and RBP4 concentrations. The results of this study may contribute to future cardiovascular risk assessments in living donors.

## Results

### Comparison of clinical parameters of transplant donors and recipients

The clinical parameters of allograft donors and recipients at baseline as well as 6 weeks and 6 months after LDKT are presented in Table 1. The age presented in Table 1 is the median age of donors (46 years) and recipients (42 years) at baseline and did not differ between both groups.

**Table 1:** Clinical parameters of donors and recipients at baseline, 6 weeks and 6 months after living-donor kidney transplantation (LDKT)^#^

	Donors			Recipients		
**N (m/f)**	**20 (6/14)**			**20 (14/6)**		
**Age (a)**	**46 (27-66)**			**42 (6-77)**		

	**Baseline**	**6 weeks after LDKT**	**6 months after LDKT**	**Baseline**	**6 weeks after LDKT**	**6 months after LDKT**

eGFR (mL/min per 1.73 m^2^)	84 (68-139)^a^***	58 (40-94)^b^	57 (40-88)^b^	8 (5-16)^A^	61 (24-145)^B^	63 (19-172)^B^
Proteinuria (mg/L)	47 (40-260) ***	40 (40-92) *	44 (40-105) *	544 (42-1943)	175 (37-762)	73 (45-1238)
UP/UC	0.09 (0.04-0.84) ***	0.10 (0.04-0.27)	0.09 (0.04-0.17)	0.78 (0.05-4.43)^A^	0.13 (0.06-0.37)^B^	0.10 (0.03-1.44)^B^
CRP (mg/dL)	0.17 (0.04-2.13)	0.34 (0.03-1.21)	0.28 (0.04-2.72)	0.21 (0.02-1.45)	0.26 (0.02-3.91)	0.22 (0.02-2.57)
Total cholesterol (mg/dL)	182 (141-279)	177 (142-289)	193 (118-269)	182 (126-353)^A^	193 (90-291)^AB^	191 (134-312)^B^
LDL cholesterol (mg/dL)	116 (67-214)	115 (72-211)	126 (68-188)	120 (76-240)	131 (72-222)	126 (72-204)
HDL cholesterol (mg/dL)	58 (39-94) **	53 (36-90)	56 (35-92)	46 (29-71)^A^	52 (36-119)^B^	50 (30-78)^B^
Trigylcerides (mg/dL)	94 (46-298) ***	124 (64-292) **	116 (72-397) *	230 (81-671)	207 (81-443)	179 (81-545)
ROH (μg/mL)	0.61 (0.37-0.84)^a^***	0.81 (0.41-1.07)^b^	0.67 (0.43-1.09)^b^	1.46 (0.67-2.25)^A^	0.88 (0.38-1.79)^B^	1.01 (0.32-1.44)^B^
RBP4 (μmol/L)	1.76 (1.08-2.13)^a^***	2.11 (1.02-3.38)^b^	2.55 (1.19-3.89)^c^	6.57 (3.19-8.77)^A^	2.80 (1.10-6.38)^B^	3.06 (0.99-5.23)^B^
TTR (μmol/L)	2.58 (0.96-5.78) ***	3.25 (0.47-5.06)	3.08 (1.91-4.99)	5.60 (2.06-8.37)^A^	3.51 (1.58-8.24)^B^	4.53 (1.05-6.38)^B^
apoRBP4 (%)	0.0 (0.0-8.2)^a^***	8.3 (0.6-13.1)^b^	7.6 (0.3-9.6)^b^	30.7 (1.2-47.6)^A^	12.9 (0.8-23.4)^B^	10.2 (0.6-31.0)^B^

^# ^Data are presented as median (range). Abbreviations used: CRP, C-reactive protein; eGFR, estimated glomerular filtration rate; HDL, high density lipoprotein; LDL, low density lipoprotein; LDKT, living-donor kidney transplantation; m/f; male/female; UP/UC, urine protein/urine creatinine ratio. Different superscripts ^a, b ^and ^c ^indicate significant differences between the different time points for donors. Different superscripts ^A ^and ^B ^indicate significant differences between the different time points for recipients. Superscripts *, ** and *** indicate significant differences between donors and recipients at the particular time point with * p < 0.05, ** p < 0.01 and *** p < 0.001.

At baseline, allograft recipients revealed significantly lower estimated GFR (eGFR, p < 0.001) in comparison to living-kidney donors. However, eGFR did not differ 6 weeks and 6 months after LDKT, respectively. Additionally, proteinuria was significantly higher in recipients than in donors at baseline (p < 0.001) as well as 6 weeks (p = 0.018) and 6 months (p = 0.013) after LDKT. Likewise, the urine protein/urine creatinine ratio (UP/UC) was higher in recipients than in donors at baseline (p < 0.001), but not 6 weeks and 6 months after LDKT, respectively. The triglyceride concentration was also significantly higher in recipients than in donors at baseline (p < 0.001), 6 weeks (p = 0.001) and 6 months (p = 0.011) after LDKT. In contrast, HDL cholesterol concentration was lower in recipients than in donors only at baseline (p = 0.001), but not 6 weeks and 6 months after LDKT. The serum concentrations of total and LDL cholesterol as well as C-reactive protein (CRP) did not differ at any time between recipients and donors.

### Changes in clinical parameters in kidney transplant donors and recipients after LDKT

In donors the unilateral nephrectomy resulted in a significant decrease of eGFR within 6 weeks after LDKT (p < 0.001) to a median eGFR of 58 mL/min per 1.73 m^2 ^and approximately 65% of donors revealed a eGFR below 60 mL/min per 1.73 m^2 ^corresponding to CKD stage 3 according the K/DOQI guidelines [17]. Six months after LDKT the eGFR was still decreased (57 mL/min per 1.73 m^2^) in comparison to baseline levels (84 mL/min per 1.73 m^2^, p = 0.001) and approximately 62% of the donors still revealed a eGFR below 60 mL/min per 1.73 m^2^. With regard to the other clinical parameters, no differences could be observed between baseline, 6 weeks and 6 months after LDKT (Table 1).

In allograft recipients transplantation resulted in an increase of eGFR within 6 weeks (61 mL/min per 1.73 m^2^) in comparison to baseline (8 mL/min per 1.73 m^2^) (p < 0.001) and the eGFR remained unchanged in the recipients for at least 6 months (53 mL/min per 1.73 m^2^). In addition, UP/UC was decreased in recipients 6 weeks after kidney transplantation in comparison to baseline (p = 0.008) and remained decreased also 6 months after transplantation (p = 0.05). Proteinuria tended to decrease in recipients during the follow-up period (544 mg/L at baseline to 73 mg/L 6 months after LDKT); however this tendency was not significant. The HDL serum concentration increased 6 weeks after transplantation (p = 0.003) in comparison to baseline and remained increased also 6 months after LDKT (p = 0.013). Finally, there were no changes in the serum concentration of CRP, total and LDL cholesterol and triglycerides during the follow-up period in allograft recipients (Table 1).

### Changes in ROH, RBP4, apoRBP4 and TTR in kidney transplant donors and recipients during follow-up period

Changes in the serum concentration of ROH, RBP4 and TTR and relative amounts of apoRBP4 before and after LDKT are shown in Table 1. At baseline, the ROH, RBP4, apoRBP4 and TTR serum concentrations were significantly higher in serum of recipients than in serum of donors (p < 0.001, for all). However, there were no differences in the parameters between donors and recipients 6 weeks and 6 months after LDKT.

For donors the unilateral nephrectomy resulted in a significant increase of ROH and RBP4 serum concentration within 6 weeks after LDKT (0.81 μg/mL with p = 0.004 and 2.11 μmol/L with p < 0.001, respectively) in comparison to baseline (0.61 μg/mL and 1.76 μmol/L, respectively). The serum concentrations of ROH and RBP4 remained increased also 6 months after LDKT (0.67 μg/mL with p = 0.001 and 2.55 μmol/L with p < 0.001, respectively). In contrast, the TTR concentration did not change significantly in serum of donors during the follow-up period (baseline 2.58 μmol/L, 6 weeks after LDKT 3.25 μmol/L, and 6 months after LDKT 3.08 μmol/L). With regard to allograft recipients, the ROH serum concentration decreased within 6 weeks after LDKT (1.46 μg/mL vs. 0.88 μg/mL, p < 0.001) and remained decreased also 6 months after LDKT (1.01 μg/mL, p < 0.001). In contrast, the RBP4 serum concentrations decreased within 6 weeks after LDKT from 6.57 μmol/L at baseline to 2.80 μmol/L (p < 0.001) and remained decreased in comparison to baseline also 6 months after LDKT (3.06 μmol/L with p = 0.007). Likewise, the TTR concentration in serum of recipients also decreased within 6 weeks after LDKT (3.51 μmol/L, p = 0.001) and remained decreased also 6 months after LDKT (4.53 μmol/L, p < 0.001) in comparison to baseline (5.60 μmol/L). However, even 6 months after LDKT neither ROH nor RBP4 nor TTR serum concentrations of recipients decreased to levels comparable to those of donors at baseline.

With regard to apoRBP4, allograft recipients revealed a significantly higher relative amount of apoRBP4 than donors at baseline (p < 0.001), but there were no more differences 6 weeks as well as 6 months after LDKT. The unilateral nephrectomy resulted in a significant increase of the relative amount of apoRBP4 in serum of donors 6 weeks after LDKT (8.3% vs. 0% at baseline, p = 0.002), which remained increased for at least 6 months after LDKT (7.6%) in comparison to baseline (p = 0.002). Vice versa, the relative amount of apoRBP4 decreased in serum of recipients within 6 weeks after LDKT (12.9% vs. 30.7% at baseline, p = 0.001) and did not change for at least 6 months after LDKT (10.2%) in comparison to baseline values (p = 0.001).

### Correlation of eGFR with ROH, RBP4, apoRBP4 and TTR in donors and recipients

**Figure 1:**
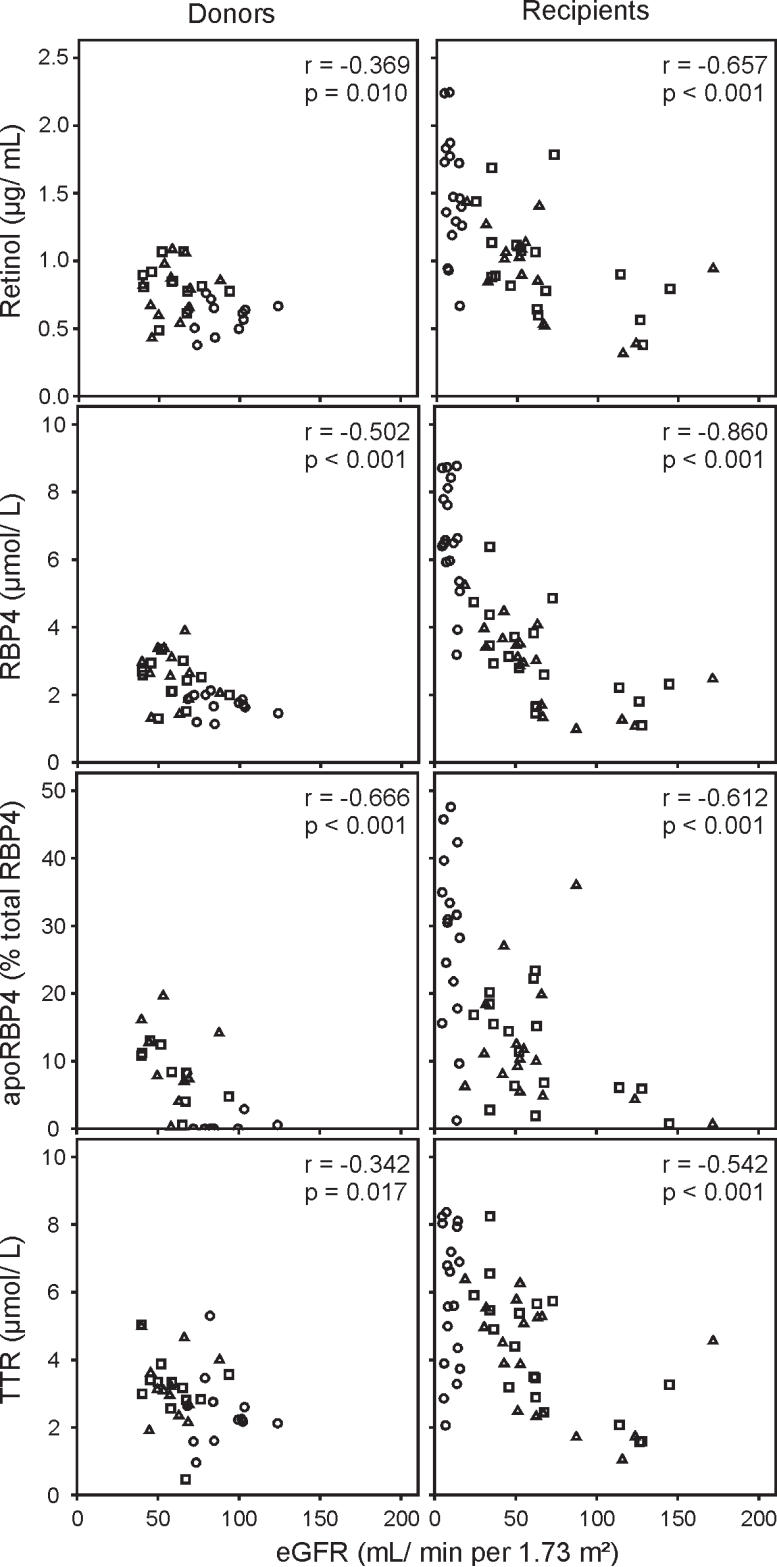
**Correlation of retinol, RBP4, apoRBP4 and TTR with eGFR of donors and recipients represented as pooled values of measurements at baseline (circles), 6 weeks (squares) and 6 months (triangles) after LDKT^#^**. ^# ^Data are presented as Spearman-Rho rank correlation coefficients. Abbreviations used: eGFR, estimated glomerular filtration rate; LDKT, living-donor kidney transplantation; RBP4, retinol-binding protein 4; TTR, transthyretin.

To evaluate the association of eGFR with the components of the vitamin A transport complex and the relative amount of apoRBP4 data at baseline, 6 weeks and 6 months after LDKT were pooled for donors and recipients separately. Non-parametric correlation analyses were performed for each data set (donors and recipients) independent from each other and scatter plots as well as Spearman Rho correlation coefficients (r) are presented in Figure 1.

For both, donors and allograft recipients, a significant inverse correlation of eGFR with ROH, RBP4 and TTR serum concentration as well as relative amounts of apoRBP4 could be detected. Thereby, donors revealed particularly strong inverse associations of eGFR with RBP4 serum concentration (r = -0.502, p < 0.001) and with apoRBP4 (r = -0.666, p < 0.001). With regard to allograft recipients, all four parameters were highly inversely associated with eGFR (p < 0.001), however, RBP4 serum concentration revealed the strongest correlation (r = -0.860, p < 0.001).

## Discussion

The kidneys play an important role in the metabolism of vitamin A and its transport proteins [5,6,18] and it is well documented that a reduced kidney function due to acute or chronic renal failure is associated with increased serum concentrations of ROH, RBP4 and TTR [5,8,9]. However, very little is known about changes in the vitamin A transport complex in serum of donors after LDKT, which represents a sudden loss of kidney function. Therefore, we investigated the concentrations of ROH, RBP4 and TTR in serum of donors and the respective recipients before and after transplantation.

The main clinical consequence of unilateral nephrectomy in donors was a decrease in eGFR to approximately 65% of pre-nephrectomy values, which remained decreased also 6 months after LDKT as also described by others before [19-21]. In allograft recipients, LDKT resulted in a general improvement of kidney function, indicated by an increase of eGFR and decrease of UP/UC as expected and confirming previous results of others [22-25].

In donors the ROH and RBP4 serum concentrations as well as relative amounts of apoRBP4 (RBP4 unbound to ROH) increased post-nephrectomy and remained increased during the follow-up period of 6 months. In addition, it could be demonstrated that the increase of all three parameters was paralleled by a decrease of the donor's eGFR and correlation analysis revealed a significant inverse association of eGFR with ROH, RBP4 and apoRBP4, respectively. These results confirmed and expanded the observations of Argiles et al. [26], who reported an increase of ROH and RBP4 serum concentration in donors seven days after LDKT.

The increase of ROH and RBP4 serum concentration and relative amounts of apoRBP4 is most likely explained by the essential role of the kidneys in vitamin A metabolism. Under physiological conditions the complex of ROH, RBP4 and TTR ensures the transport of vitamin A in the circulation. After the delivery of ROH to the target tissue, the remaining complex of apoRBP4 and TTR dissociates and the resulting free apoRBP4 is then filtered and degraded in the kidneys [5,7]. Therefore, a reduction of kidney function is associated with an increase of apoRBP4 in serum [27,28], as also seen in the donors of the present study. In this context, Gerlach and Zile [29] proposed, that apoRBP4 provides a positive feedback signal for the hepatic release of holoRBP4 (RBP4 in complex with ROH), resulting in an increase of ROH and an additional increase of RBP4 serum concentration. Therefore, the increased concentrations of ROH and RBP4 in serum of donors of the present study are most likely attributed to the decreased kidney function and the resulting increase of apoRBP4 may in turn trigger the increased secretion of holoRBP4 from the liver contributing the increase of ROH and RBP4 serum concentration. The inverse association of eGFR with ROH, RBP4 and apoRBP4 in the present study supports this hypothesis and emphasizes the importance of the kidneys in homeostasis of the vitamin A metabolism. However, these results also indicate that in donors the remaining kidney function after LDKT does not seem to be adequate to maintain a normal vitamin A metabolism.

The fact that TTR serum concentration of donors does not seem to be affected by nephrectomy in the present study underlines the lesser importance of kidneys to TTR metabolism [6]. Nevertheless, although not significant, the TTR concentration in serum of donors tended to increase and a longer follow-up period may might have resulted in a significant increase of TTR as well.

In view of the vitamin A transport complex, the allograft recipients revealed a reduction of ROH, RBP4, and TTR serum concentration as well as apoRBP4 within 6 months after LDKT, which is in accordance to results previously reported by Kelleher et al. [30]. The decrease in serum concentration of all four compounds was paralleled by an increase of eGFR and may be mainly explained by the restoration of kidney function by LDKT. Nevertheless, even 6 months after LDKT none of the components of the vitamin A transport complex decreased to values comparable to those of donors before LDKT, which emphasize the incomplete remission of kidney function with regard to vitamin A metabolism as suggested by Kelleher et al. [30].

The elevated concentrations of ROH and RBP4 in serum of donors after LDKT raise the question concerning the possible consequences. In principle, kidney donation has been associated with a low risk for donors with regard to surgery-associated as well as long-term complications such as hypertension, CKD, and overall mortality [1-3,31]. However, evidence is accumulating that incipient renal failure indicated by a reduced eGFR is strongly associated with an increased risk for CVD [4]. In fact, recent data indicate that the long-term risks associated with living-kidney donation are higher than previously thought [1] and that there is indeed a paucity of data concerning cardiovascular risk factors in donors after LDKT [32]. The situation is exacerbated by the trend of accepting donors beyond the traditional rigorous inclusion criteria due to the increasing demand for kidney transplantations [32].

In this context, the increased concentrations of ROH and RBP4 in serum of donors might contribute to the risk of CVD, since elevated levels of both parameters have been associated with CVD [12,13]. Furthermore, high ROH and RBP4 serum concentrations have been linked to increased intima-media thickness [14,15] and RBP4 serum concentration to a higher fat content in vessel walls and atherosclerotic plaques [14]. The underlying mechanisms for these associations remain to be elucidated, but lipid modulating activities of retinoids and retinol-binding proteins have been suggested as an important factor [14,33].

In addition, an elevated serum concentration of RBP4 has been linked to insulin resistance [16] as well as subclinical inflammation [34]. Thus, the nephrectomy-associated increase of RBP4 serum concentrations may explain the increased susceptibility of living-kidney donors for insulin resistance as "described by Shehab-Eldin et al. [35]. However, since the present study was an observational study, which primary aim was to analyze the nephrectomy-associated changes in ROH, RBP4 and TTR in living-kidney donors, the association of the vitamin A transport complex with CVD and insulin resistance remains speculative and should be a matter of future research.

## Conclusion

In summary, unilateral nephrectomy resulted in an increase of ROH and RBP4 serum concentration in living kidney donors. This increase remained during the follow-up period of 6 months, indicating that the capacity of the remaining kidney is not adequate to balance the vitamin A metabolism in donors. Since high levels of ROH and RBP4 have been associated with an increased risk of CVD and insulin resistance, future research should focus on these associations in donors especially with regard to the baseline risk for CVD and insulin resistance as well as on potential underlying mechanisms.

## Materials and methods

### Subjects and study design

A total of 20 kidney allograft recipients and the respective donors were recruited at the Medical Department, Division of Nephrology and Intensive Care Medicine and the Department of Pediatric Nephrology, both Charité Universitätsmedizin Berlin. The group of allograft recipients consisted of 17 adult (12 male and 5 female) and 3 pediatric (2 male and 1 female) patients. The group of donors comprised 6 adult men and 14 adult women. Fifty-five per cent of the donors were related to the respective recipients. After transplantation, allograft recipients received corticosteroids (prednisolone or methylprednisolone (n = 20), tacrolimus (n = 14), mycophenolate mofetil (n = 17), and basiliximab (n = 2)) and cyclosporine A (n = 5) for immunosuppression.

Blood samples of donors and recipients were drawn after an overnight fast 1 to 2 days before LDKT (baseline) as well as 6 weeks and 6 months after LDKT. The blood samples were centrifuged for serum preparation and aliquots were stored at -80°C until assayed. The study design was approved by the local ethics committee and written informed consent was obtained before the study from all participants and parents of the pediatric patients, respectively.

### Measurement of clinical parameters

Blood was taken during routine appointments, creatinine, protein, total, LDL and HDL cholesterol, triglycerides, and CRP were measured by routine laboratory techniques (and analyzer) in serum. GFR was calculated based on creatinine levels using the 4-variables-MDRD equation for adult and Schwartz formula for pediatric patients [17], respectively.

### Determination of ROH, RBP4, TTR, and apoRBP4

For quantification of ROH in serum a modified gradient reversed-phase high-performance liquid chromatography (rp-HPLC) system (Waters, Germany) was used after organic extraction as previously described in detail [36]. RBP4 and TTR serum concentrations were determined by non-commercial enzyme-linked immunosorbent assays (ELISA) as described elsewhere [37,38]. For both ELISA a standard was used containing RBP4 and TTR obtained from human blood, respectively (Dade Behring, Germany).

The relative amount of apoRBP4 (% of total RBP4) was determined by native polyacrylamide gel electrophoresis with subsequent immunoblotting as described by Frey et al. in detail [39].

### Statistical analysis

The results are expressed as median and ranges except otherwise stated. The statistical analysis was performed using PASW statistics version 17.0. Differences between donors and recipients were analyzed by Mann-Whitney U rank sum test. The Friedman test for related variables was used to test for significant differences between the different time points (baseline, 6 weeks and 6 months after LDKT) for donors and recipients separately. If there was a significant effect, Wilcoxon rank test was performed to describe differences between two time points. For calculation of correlation coefficients data at baseline, 6 weeks and 6 months after LDKT were cumulated for donors and recipients separately and Spearman-Rho rank correlation test was performed for both pooled data sets independent from each other. Values of P less than 0.05 were considered to be statistically significant (two-tailed).

## List of abbreviations

CKD: chronic kidney disease; CRP: C-reactive protein; CVD: cardiovascular disease; eGFR: estimated glomerular filtration rate; K/DOQI: kidney disease outcome quality initiative; LDKT: living-donor kidney transplantation; MDRD: modification of diet in renal disease; RBP4: retinol-binding protein 4; ROH: retinol; TTR: transthyretin; UP/UC: urine protein/urine creatinine ratio.

## Competing interests

The authors declare that they have no competing interests.

## Authors' contributions

AH carried out determination of ROH, RBP4 and TTR as well as statistical analysis and interpretation of data and drafted the manuscript. JR contributed to data analysis and interpretation, helped to draft the manuscript and revised the manuscript critically. CK participated in acquisition of data and statistical analysis and revised the manuscript critically. PR conceived the study and participated in its coordination. AS participated in coordination of the study, collection of samples and data acquisition. UQ participated in conception of the study design and revised the manuscript critically for important intellectual content. FJS revised the manuscript critically and provided important intellectual content. All authors read and approved the final manuscript.
